# NLRP3 Deficiency Attenuates Renal Fibrosis and Ameliorates Mitochondrial Dysfunction in a Mouse Unilateral Ureteral Obstruction Model of Chronic Kidney Disease

**DOI:** 10.1155/2017/8316560

**Published:** 2017-02-28

**Authors:** Honglei Guo, Xiao Bi, Ping Zhou, Shijian Zhu, Wei Ding

**Affiliations:** ^1^Division of Nephrology, The Fifth People's Hospital of Shanghai, Fudan University, 128 Ruili Road, Shanghai 200240, China; ^2^Division of Nephrology, Shanghai Ninth People's Hospital, School of Medicine, Shanghai Jiaotong University, 639 Zhizaoju Road, Shanghai 200011, China; ^3^Department of Pediatrics, The 2nd Hospital of Harbin Medical University, Harbin 150086, China

## Abstract

*Background and Aims.* The nucleotide-binding domain and leucine-rich repeat containing PYD-3 (NLRP3) inflammasome has been implicated in the pathogenesis of chronic kidney disease (CKD); however, its exact role in glomerular injury and tubulointerstitial fibrosis is still undefined. The present study was performed to identify the function of NLRP3 in modulating renal injury and fibrosis and the potential involvement of mitochondrial dysfunction in the murine unilateral ureteral obstruction (UUO) model of CKD.* Methods.* Employing wild-type (WT) and NLRP3^−/−^ mice with or without UUO, we evaluated renal structure, tissue injury, and mitochondrial ultrastructure, as well as expression of some vital molecules involved in the progression of fibrosis, apoptosis, inflammation, and mitochondrial dysfunction.* Results.* The severe glomerular injury and tubulointerstitial fibrosis induced in WT mice by UUO was markedly attenuated in NLRP3^−/−^ mice as evidenced by blockade of extracellular matrix deposition, decreased cell apoptosis, and phenotypic alterations. Moreover, NLRP3 deletion reversed UUO-induced impairment of mitochondrial morphology and function.* Conclusions.* NLRP3 deletion ameliorates mitochondrial dysfunction and alleviates renal fibrosis in a murine UUO model of CKD.

## 1. Introduction

Chronic kidney disease (CKD) is complicated progressive disease that is becoming an increasingly important public health issue worldwide [1, 2]. A comprehensive understanding of the mechanisms underlying the progression of CKD is required. Recent studies have shown a close relationship between persistent inflammation and development of various forms of CKD [3, 4], indicating that this issue merits further investigation.

Inflammation responses are induced by both infection and tissue damage to restore homeostasis. Pattern recognition receptors (PRRs) play a primary role in this process by recognizing both intra- and extracellular danger signals [5]. Nucleotide-binding domain and leucine-rich repeat containing PYD-3 (NLRP3) is one of four confirmed inflammasome-forming PRRs identified to date. After formation and activation, the NLRP3 inflammasome associates with the adaptor protein apoptosis speck-like protein containing a caspase recruitment domain (ASC) via its pyrin domain. ASC then associates with procaspase 1 in an interaction which induces the autocatalytic cleavage and activation of procaspase 1. Activated caspase 1 cleaves pro-IL-1*β* and pro-IL-18 to generate the mature, secreted forms of these cytokines [6]. Recent reports have demonstrated that the NLRP3 inflammasome is involved in the pathogenesis of CKD [7–10]. Our previous study also showed that NLPR3 inflammasome activation contributes to aldosterone-induced renal tubular injury [11].

In addition to the role of inflammation, mitochondrial dysfunction (MTD) is also implicated as an important factor in the development of CKD [12–14]. Mitochondria are important organelles involved in cellular energy production and metabolism. MTD is associated with increased oxidative stress and inflammation [15] and may also result in reduced ATP production, increased reactive oxygen species (ROS) generation, and release of proapoptotic products, such as mitochondrial DNA (mtDNA) and cytochrome *c*. Although our recent study demonstrated that inhibition of mitochondrial complex I significantly attenuated MTD, and the inflammasome response in aldosterone-infused rats [16], the role of the NLRP3 inflammasome in modulating mitochondrial function in a murine unilateral ureteral obstruction (UUO) model of CKD disease is still unclear.

In this study, we investigated the contribution of NLRP3 inflammasome activation to MTD and loss of kidney structure and function in a murine UUO model of CKD.

## 2. Materials and Methods

### 2.1. Materials

Antibodies of the following specificities were used in this study: caspase 3, *α*-SMA, and E-cadherin (Cell Signaling Technology); IL-1*β*, IL-18, collagen I, and fibronectin (Abcam); caspase 1 and PGC-1a (Santa Cruz Biotechnology); and *β*-actin (Sigma–Aldrich).

### 2.2. Animal Studies

All animal experiments were performed with the approval of the Animal Care Committee at Jiao Tong University. C57BL/6J (wild-type, WT) mice were purchased from Shanghai SLAC Laboratory Animals (Shanghai, China). NLRP3^−/−^ mice (C57BL/6J genetic background) were purchased from the Jackson Laboratory (Sacramento, CA). Only age and sex-matched mice were used in experiment. NLRP3 deficiency was genetically confirmed by RT-PCR screening. Male WT and NLRP3^−/−^ mice (aged 8–10 weeks; *n* = 6 per group) underwent left ureteral ligation as described previously [17]. Complete ureteral obstruction was produced by double ligation with 4-0 silk thread. These mice comprised the WT/UUO and NLRP3^−/−^/UUO groups, respectively. Comparable groups of male mice underwent an identical procedure without ligation as the WT/Sham group and NLRP3^−/−^/Sham groups (*n* = 6 per group). Mice were sacrificed on days 1, 3, 7, and 14 after UUO. At the end of the experiment, kidney samples were collected and immediately frozen in liquid nitrogen for storage at −80°C.

### 2.3. Histological and IHC Studies

Samples of kidney tissue were sectioned (thickness 4 *μ*m), processed, and stained with periodic acid-Schiff reagent (PAS) or Masson's trichrome stain according to our previous report [18]. After PAS staining, we evaluated the severity of glomerular injury in experimental mice using light microscopy in accordance with the following semiquantitative grades: grade 0, normal; grade 1, segmental lesion < 25%; grade 2, 25%–50%; grade 3, 50%–75%; grade 4, 75%–100%. At least 20 glomeruli were analyzed in each group. The area of tubulointerstitial fibrosis was evaluated in 10 random 400x magnification fields following Masson trichrome staining.

The sections were also stained with anti-collagen I (1 : 1000) or anti-fibronectin antibodies (1 : 1000) and the immunohistochemistry was quantified as described in our previous study [19]. In brief, the areas of positive collagen I and fibronectin staining were expressed as a percentage of the total area determined using an image analyzer (Winroof).

### 2.4. Western Blot Analysis

Western blotting of renal tissue was performed as previously described [20]. After blotting, the membranes were incubated overnight with antibodies against *α*-SMA, E-cadherin, cleaved caspase 3, caspase 1, IL-18, and IL-1*β* (all diluted by Tris-buffered saline-Tween 20 (TBST) containing 5% BSA). After washing with TBST, blots were incubated with secondary antibodies for 2 h. The immune complexes were visualized with an enhanced chemiluminescent system (Amersham, Little Chalfont, Bucks., UK) and band intensity quantified using Quantity One (Bio-Rad, Hercules, CA, USA).

### 2.5. Terminal Deoxyribonucleotide Transferase (TdT-) Mediated dUTP Nick-End Labeling (TUNEL) Assay

Apoptosis in kidney tissue was detected using the TUNEL assay. Briefly, deparaffinized kidney tissue section was treated in a permeabilization solution (0.1% Triton X-100 in 0.1% sodium citrate) at 4°C for 2 min. The tissue was then washed twice with PBS and incubated with 50 *μ*L TUNEL reaction mixture for 60 min at 37°C in the dark. For quantification, 20 fields were randomly selected in each section, and the number of TUNEL-positive cells was counted in a standard area (1 mm^2^).

### 2.6. Electron Microscopy Essay

To make a detailed analysis of the ultrastructural morphology of mitochondria, kidney tissue samples were fixed with 2.5% glutaraldehyde at room temperature and then cut into 1 mm^3^ pieces using a scalpel. According to the method described in our previous report [16], we prepared ultrathin sections (60 nm) using a microtome. The sections were then placed on copper grids and stained with uranyl acetate and lead citrate for evaluation by electron microscopy.

### 2.7. Real-Time Reverse Transcription Polymerase Chain Reaction (RT-PCR)

Total RNA was isolated from kidney tissue obtained from each group and reverse-transcribed to cDNA. RT-PCR analysis was performed according to a previously described protocol [21] using the cDNA as a template and the following primers: mtDNA: forward 5′-TTTTATCTGCATCTGAGTTTAATCCTGT-3′ and reverse 5′-CCACTTCATCTTACCATTTATTATCGC-3′; ATP synthase: forward 5′-TCCATCAAAAACATCCAGAAAA-3′ and reverse 5′-GAGGAGTGAATAGCACCACAAA-3′; ND1: forward 5′-ATCCTCCCAGGATTTGGAAT-3′ and reverse 5′-ACCGGTAGGAATTGCGATAA-3′; 18S: forward 5′-TTCGGAACTGAGGCCATGATT-3′ and reverse 5′-TTTCGCTCTGGTCCGTCTTG-3′.

### 2.8. Statistical Analyses

Data are expressed as the mean ± standard error of the mean (SEM). Comparisons between groups were performed with one-way analysis of variance (ANOVA) followed by Dunnett's multiple comparison tests or Student's *t*-test. *P* < 0.05 was considered statistically significant.

## 3. Results

### 3.1. NLRP3 Deletion Attenuates UUO-Induced Renal Injury

**Figure 1:**
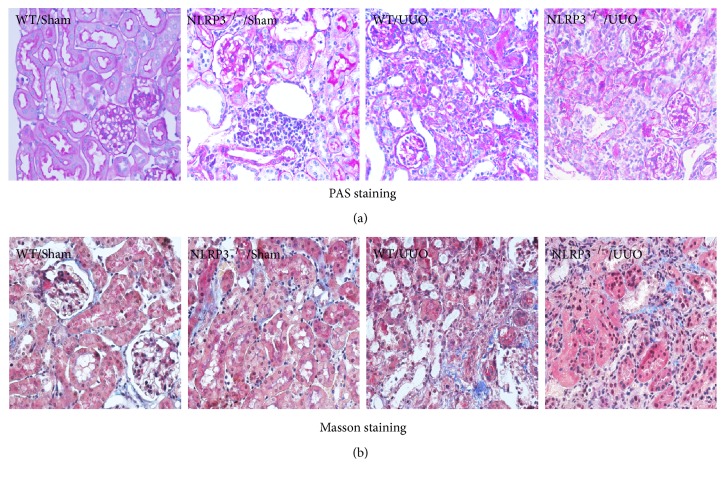
Demonstration of kidney histopathology at day 14 UUO. Representative photomicrographs of periodic acid-Schiff-stained kidney sections ((a), magnification, ×400) and Masson trichrome-stained kidney sections ((b), magnification, ×400).

Renal histopathology was examined following PAS staining and Masson staining (Figures 1(a) and 1(b), resp.). UUO caused severe glomerular injury (3.06 ± 0.12) in WT mice (*P* < 0.05, versus WT/Sham group). Compared with the WT/UUO group, the glomerular injury score was significantly decreased in the NLRP3^−/−^/UUO group (*P* < 0.05; Figures 1(a) and 3(a)). Similarly, significant tubulointerstitial fibrosis was induced by UUO in WT mice and compared with the WT/UUO group, the tubulointerstitial fibrosis index was significantly reduced in NLRP3^−/−^/UUO group (*P* < 0.05; Figures 1(b) and 3(b)).

### 3.2. NLRP3 Deletion Attenuates Renal Collagen I Deposition and Fibrosis in UUO Mice

**Figure 2:**
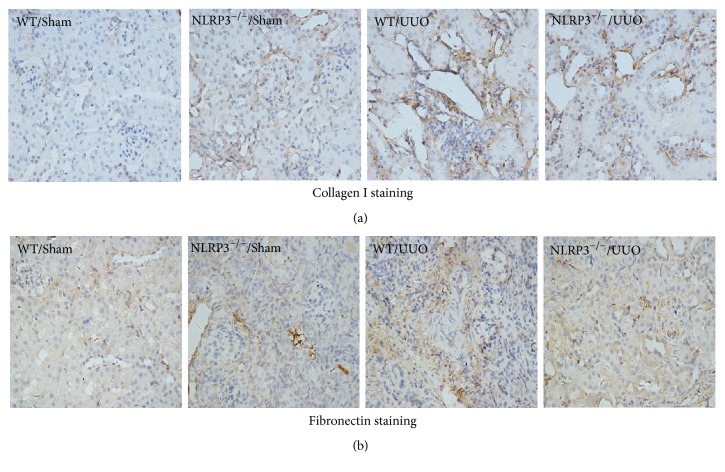
Immunohistochemical staining for deposition of collagen I ((a) magnification, ×400) and fibronection ((b), magnification, ×400) in kidney sections from different groups at day 14 after UUO.

**Figure 3:**
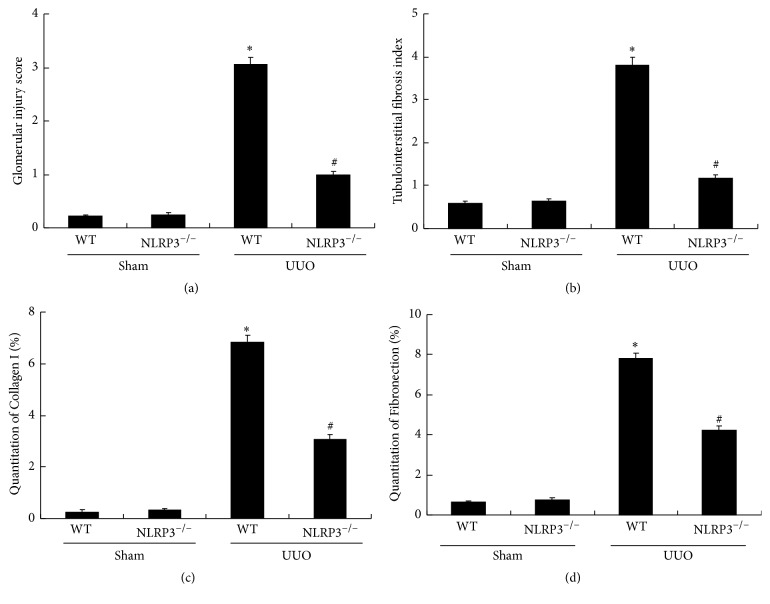
NLRP3 deletion attenuates UUO-induced glomerular injury and tubulointerstitial fibrosis as well as deposition of collagen I and fibronectin. Glomerular injury score (a) and tubulointerstitial fibrosis index (b) are evaluated according to PAS staining and Masson staining. Semiquantitative analysis of collagen I (c) and fibronectin (d) positive areas is performed according to immunohistochemical staining. Data represent the mean ± SEM (*n* = 6). ^*∗*^*P* < 0.05, WT/Sham group versus WT/UUO group; ^#^*P* < 0.05, NLRP3^−/−^/UUO group versus WT/UUO group.

Immunohistochemical analysis of collagen I and fibronectin in renal tissue sections (Figures 2, 3(c), and 3(d)) showed a significant increase in collagen I-positive and fibronectin-positive cells following UUO in WT mice. Partial but significant reversal of these effects was observed in the NLRP3^−/−^/UUO group (*P* < 0.05; Figures 2, 3(c), and 3(d)).

### 3.3. UUO-Induced Fibrosis-Related Phenotypic Alterations and Apoptosis Are Reduced by NLRP3 Deletion

**Figure 4:**
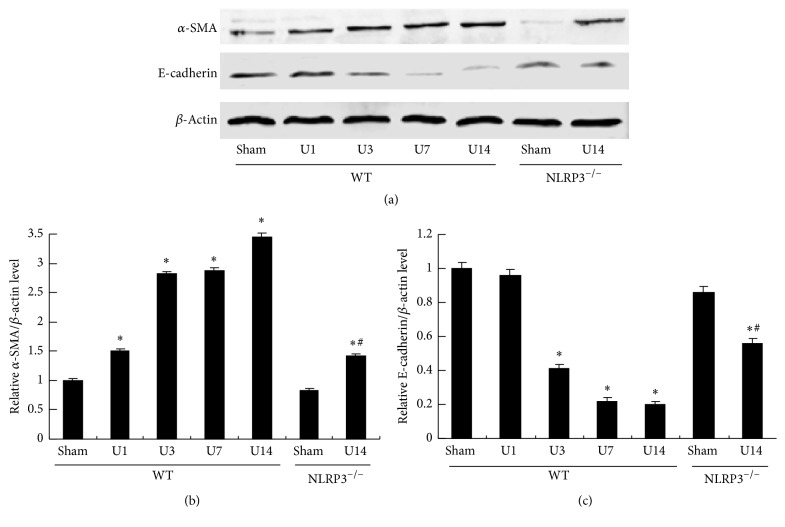
NLRP3 deletion decreases UUO-induced phenotypic alterations in UUO mice. Expression of *α*-SMA and E-cadherin protein detected in kidney samples from different groups (a). Semiquantitative analysis of *α*-SMA expression (b) and E-cadherin expression (c) normalized against *β*-actin. Data represent the mean ± SEM (*n* = 6). ^*∗*^*P* < 0.05 compared with WT/Sham group, ^#^*P* < 0.05, NLRP3^−/−^/U14 group versus WT/U14 group. U1, U3, U7, and U14 indicate that mice were sacrificed on days 1, 3, 7, and 14 after UUO.

*α*-SMA is regarded as a fibroblast marker and renal epithelial cells lose E-cadherin expression during renal fibrosis. Therefore, we examined the expression of *α*-SMA and E-cadherin in renal tissue (Figure 4). Expression of *α*-SMA protein was significantly increased in time-dependent manner after UUO induction in WT mice (*P* < 0.05) and this increase was significantly decreased in the NLRP3^−/−^ mice at day 14 after UUO (*P* < 0.05, versus WT/U14 group). In contrast, E-cadherin protein expression was significantly reduced in time-dependent manner after UUO in WT mice (*P* < 0.05). Partial but significant reversal of these effects was observed in the NLRP3^−/−^/UUO group (*P* < 0.05, versus WT/U14 group).

**Figure 5:**
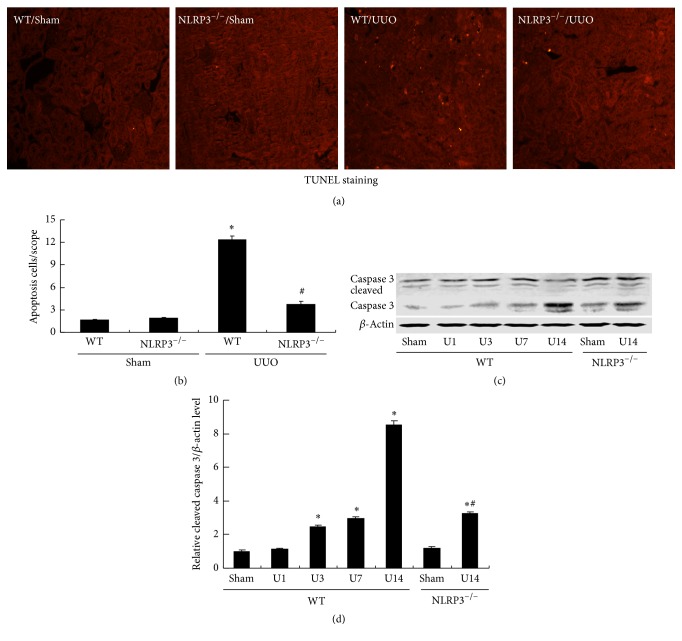
NLRP3 deletion ameliorates UUO-induced cell apoptosis in UUO mice. Representative images of TUNEL staining are shown ((a), magnification, ×200). Bar graph indicates the mean number of TUNEL-positive tubular cells per field (b). Expression of caspase 3 protein was detected in kidney samples (c). Semiquantitative analysis of cleaved caspase 3 normalized against *β*-actin (d). Data represent the mean ± SEM (*n* = 6). ^*∗*^*P* < 0.05, compared with WT/Sham group, ^#^*P* < 0.05, NLRP3^−/−^/U14 group versus WT/U14 group. U1, U3, U7, and U14 indicate that mice were sacrificed on days 1, 3, 7, and 14 after UUO.

The effects of UUO on apoptosis were evaluated by TUNEL staining and detection of caspase 3 protein by Western blot analysis (Figure 5). TUNEL staining revealed markedly more apoptotic cells in the WT/UUO group compared with the WT/Sham group and this UUO-induced apoptosis was evidently inhibited by NLRP3 deletion (Figures 5(a) and 5(b)). This was confirmed by Western blot analysis of caspase 3. Cleavage of caspase 3 to generate the activated protein increased significantly after UUO in WT mice (*P* < 0.05); this increase was significantly decreased in the NLRP3^−/−^ mice at day 14 after UUO (*P* < 0.05, versus WT/U14 group) (Figures 5(c) and 5(d)).

### 3.4. NLRP3 Deletion Ameliorates UUO-Induced Expression of Inflammatory Cytokines

**Figure 6:**
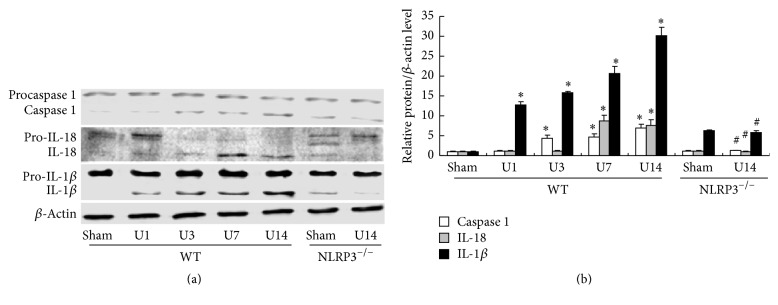
NLRP3 deletion decreases UUO-upregulated expression of NLRP3 inflammasome-regulated cytokines. Expression of caspase 1, IL-1*β*, and IL-18 in kidney samples was detected (a). Semiquantitative analysis of caspase 1, IL-1*β*, and IL-18 normalized against *β*-actin (b). Data represent the mean ± SEM (*n* = 6). ^*∗*^*P* < 0.05, compared with WT/Sham group, ^#^*P* < 0.05, NLRP3^−/−^/U14 group versus WT/U14 group. U1, U3, U7, and U14 indicate that mice were sacrificed on days 1, 3, 7, and 14 after UUO.

The expression of NLRP3 inflammasome-regulated inflammatory cytokines was evaluated by Western blot analysis (Figures 6(a) and 6(b)). At day 14 after UUO, significant increases in renal expression of caspase 1 (7.15-fold), IL-1*β* (29.8-fold), and IL-18 (7.42-fold) proteins were detected in WT mice (all *P* < 0.05 versus WT/Sham group) and these effects were significantly inhibited in the NLRP3^−/−^ mice at day 14 after UUO (all *P* < 0.05, versus WT/U14 group).

### 3.5. NLRP3 Deletion Attenuates UUO-Induced Mitochondrial Dysfunction

**Figure 7:**
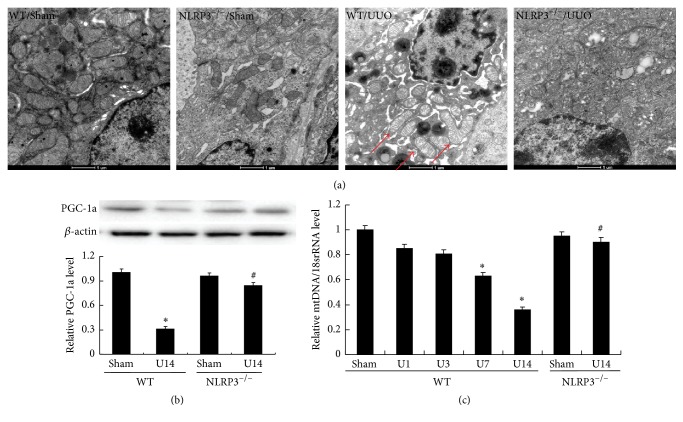
NLRP3 deletion reverses UUO-induced mitochondrial dysfunction. Representative electron microscopy photomicrographs of ultrastructural morphology of mitochondria (a), (magnification ×12,000). Arrow indicates swollen mitochondria. Expression of PGC-1a in kidney sample was detected, and semiquantitative analysis of PGC-1a normalized against *β*-actin (b). Semiquantitative analysis of mtDNA expression normalized against 18S performed by real-time PCR (c). Data represent the mean ± SEM (*n* = 6). ^*∗*^*P* < 0.05, compared with WT/Sham group, ^#^*P* < 0.05, NLRP3^−/−^/U14 group versus WT/U14 group. U1, U3, U7, and U14 indicate that mice were sacrificed on days 1, 3, 7, and 14 after UUO.

**Figure 8:**
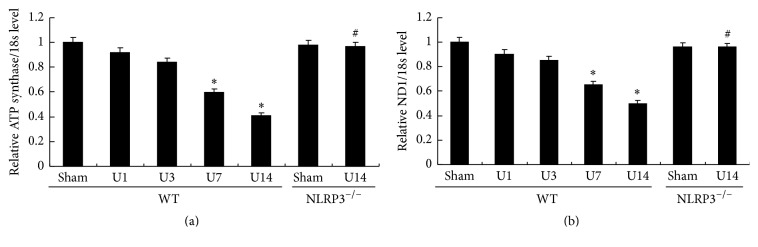
NLRP3 deletion attenuates UUO-induced mitochondrial dysfunction. Semiquantitative analysis of ATP synthase (a) and ND1 (b) normalized against 18S performed by real-time PCR. Data represent the mean ± SEM (*n* = 6). ^*∗*^*P* < 0.05, compared with WT/Sham group, ^#^*P* < 0.05, NLRP3^−/−^/U14 group versus WT/U14 group. U1, U3, U7, and U14 indicate that mice were sacrificed on days 1, 3, 7, and 14 after UUO.

MTD is characterized by disordered intracellular ATP synthesis, mtDNA damage, and excessive accumulation of reactive oxygen species in a process that is regulated by mitochondrial NADH dehydrogenase 1 (ND1). Observation of the ultrastructural morphology of renal cells of WT mice after UUO revealed swollen mitochondria with disorganized and fragmented cristae (Figure 7(a)). Moreover, these effects were accompanied by significantly decreased PGC-1a protein, mtDNA, ATP synthase, and ND1 at day 14 in the WT/UUO group compared with the WT/Sham group (all *P* < 0.05; Figures 7 and 8). The decreased levels of PGC-1a protein, mtDNA, ATP synthase, and ND1 induced by UUO in WT mice were significantly ameliorated in the NLRP3^−/−^ mice at day 14 after UUO (all *P* < 0.05, versus WT/U14 group).

## 4. Discussion

During development of CKD, loss of renal function is closely related to renal fibrosis. Although numerous studies suggest that persistent inflammation contributes to chronic kidney injury, the underlying mechanism of inflammation-induced CKD is still unknown. The results of the present study indicate that activation of the NLRP3 inflammasome accelerates renal fibrosis through mitochondrial dysfunction in the murine UUO model of CKD. Thus, the NLPR3 inflammasome is implicated as a potential target in the prevention of renal fibrosis progression.

Inflammation is part of the normal response to tissue damage. Despite its original purpose of restoring homeostasis, overt or chronic inflammation can lead to secondary tissue damage and organ dysfunction [22]. Activation of the inflammasome and subsequent release of proinflammatory cytokines have been shown to be involved in many disease conditions [23]. NLRP3 expression was found to be increased in renal biopsy samples from patients with various renal diseases, with increased levels correlating with decreased renal function [7]. Patients with CKD also exhibited elevated levels of inflammasome-regulated cytokines, such as IL-18 [24].

The UUO model of progressive CKD is often used to investigate the mechanisms of renal fibrosis. Vilaysane et al. reported less renal inflammation and fibrosis at 14 days after UUO in the absence of NLRP3 compared with the levels detected in its presence, which was associated with the activation of the inflammasome-dependent pathway of NLRP3 [7]. Furthermore, Bani-Hani et al. showed that IL-18 neutralization prevents renal injury and fibrosis after UUO in mice [25]. In the present study, we found that activation of the NLRP3 inflammasome and its downstream cytokines were increased in a time-dependent manner after UUO in WT mice. Moreover, NLRP3 deletion significantly ameliorated UUO-induced glomerular injury and tubulointerstitial fibrosis through decreased accumulation of extracellular matrix components such as collagen I and fibronectin as well as inhibition of apoptosis and phenotypic transition of renal intrinsic cells. In addition, our results implicate the release of mature IL-1*β* and IL-18 due to NLRP3 inflammasome activation in glomerular injury and tubulointerstitial fibrosis. Taken together, these findings suggested an important role for canonical NLRP3 inflammasome activation (the NLRP3 inflammasome-caspase 1-IL-1*β*/IL-18 axis) in renal fibrosis in the murine UUO model of CKD.

MTD is closely associated with cell apoptosis and tissue injury. Recent studies have suggested that MTD is a key event in the pathogenesis of CKD [26]. In this study, we have demonstrated increasingly severe MTD in the UUO model, manifested swollen mitochondria, together with decreased mtDNA, ATP synthase, and ND1. MtDNA, which is distinct from nuclear DNA, encodes mitochondrial proteins that are essential for oxidative phosphorylation as well as production of uridine and pyruvate [27]. ND1 is one subunit of mitochondrial complex I, the loss of which leads to disruption of complex I assembly, followed by mitochondrial metabolic dysfunction [28]. ATP synthase is vital for ATP production, with loss of this enzyme resulting causing a serious deficiency in the production of energy [29]. Thus, the reductions in mtDNA, ATP synthase, and ND1 are implicated in MTD observed in the UUO model may correlate with the observed increases in glomerular injury and tubulointerstitial fibrosis.

In many reports, MTD is regarded as important contribution to activation of NLRP3 inflammasome in kidney damage [30]. Moreover, mitochondrial ROS inhibitors, mito-TEMPO, and rotenone significantly inhibited Nlrp3 inflammasome activation [11, 16]. However, other studies have suggested that the NLRP3 inflammasome mediates renal injury via impaired mitochondrial function [31, 32], which implies the existence of positive feedback regulation between activation of NLRP3 inflammasome and mitochondrial dysfunction. It has been noted that a number of proinflammatory cytokines, including TNF-*α* and IL-1*β*, may induce mitochondrial injury by decreasing the activity of complex I, production of ATP, and mitochondrial transcription, which is related to the induction of apoptosis. These cytokines also induce marked accumulation of ROS [33]. In addition, the NLRP3 inflammasome has been shown to activate caspase 1 via increased mitochondrial membrane permeability and cytosolic mtDNA release [34]. In this study, NLRP3 deletion significantly preserved kidney function via attenuating mitochondrial dysfunction evidenced by improved swollen mitochondria, ameliorated mtDNA, ATP synthase, and ND1 at 14 days after UUO. These data presented suggest that inflammasome activation in the UUO model occurs upstream of mitochondrial dysfunction, which indicates the potential role of the NLRP3 inflammasome in the induction of mitochondrial dysfunction in the UUO model.

In summary, our study demonstrates mitochondrial dysfunction and the involvement of NLRP3 inflammasome activation and subsequent proinflammatory cytokine production in glomerular injury and tubulointerstitial fibrosis in the UUO model. Moreover, NLRP3 deletion significantly decreased proinflammatory cytokine release, reversed mitochondrial dysfunction, and preserved kidney ultrastructure. These results support the concept that activation of NLRP3 inflammasome induces mitochondrial dysfunction and subsequent renal fibrosis in the murine UUO model of CKD. Our characterization of the NLRP3 inflammasome/mitochondrial dysfunction axis may offer new insights into the prevention of renal fibrosis in CKD.
